# Fine-mapping QTL for mastitis resistance on BTA9 in three Nordic red cattle breeds

**DOI:** 10.1111/j.1365-2052.2008.01729.x

**Published:** 2008-08

**Authors:** G Sahana, M S Lund, L Andersson-Eklund, N Hastings, A Fernandez, T Iso-Touru, B Thomsen, S Viitala, P Sørensen, J L Williams, J Vilkki

**Affiliations:** * Department of Genetics and Biotechnology, Faculty of Agricultural Sciences, Aarhus University, Research Centre FoulumDK-8830 Tjele, Denmark; † Department of Animal Breeding and Genetics, Swedish University of Agricultural SciencesS-750 07 Uppsala, Sweden; ‡ Roslin InstituteRoslin, Midlothian EH25 9PS, UK; § MTT, Agrifood Research Finland, Biotechnology and Food ResearchFIN-31600 Jokioinen, Finland; ¶ Parco Tecnologico Padano, via Einstein, Polo UniversitarioLodi 26900, Italy

**Keywords:** dairy cattle, mastitis, quantitative trait loci, somatic cell score

## Abstract

A QTL affecting clinical mastitis and/or somatic cell score (SCS) has been reported previously on chromosome 9 from studies in 16 families from the Swedish Red and White (SRB), Finnish Ayrshire (FA) and Danish Red (DR) breeds. In order to refine the QTL location, 67 markers were genotyped over the whole chromosome in the 16 original families and 18 additional half-sib families. This enabled linkage disequilibrium information to be used in the analysis. Data were analysed by an approach that combines information from linkage and linkage disequilibrium, which allowed the QTL affecting clinical mastitis to be mapped to a small interval (<1 cM) between the markers *BM4208* and *INRA084*. This QTL showed a pleiotropic effect on SCS in the DR and SRB breeds. Haplotypes associated with variations in mastitis resistance were identified. The haplotypes were predictive in the general population and can be used in marker-assisted selection. Pleiotropic effects of the mastitis QTL were studied for three milk production traits and eight udder conformation traits. This QTL was also associated with yield traits in DR but not in FA or SRB. No QTL were found for udder conformation traits on chromosome 9.

## Introduction

Mastitis is the most frequent disease in dairy cattle with large economic consequences (e.g. Lescourret & Coulon 1994; Schukken *et al.* 1997; Kossaibati *et al.* 1998). Therefore, improving resistance to mastitis is an important breeding objective. However, it is difficult to make appreciable genetic progress by traditional breeding methods because the heritability of the trait is low (Heringstad *et al.* 2000; Hansen *et al.* 2002; Carlén *et al.* 2004) and there is unfavourable genetic correlation with production traits (Lund *et al.* 1999; Carlén *et al.* 2004). In addition, the trait is difficult to record objectively. Resistance to mastitis is therefore a prime candidate for marker-assisted selection (MAS) and many studies have attempted to detect QTL affecting this trait. At present, there are two major barriers to using MAS for improving resistance to mastitis. First, most studies have identified QTL for somatic cell score (SCS) and not for clinical mastitis (CM). Somatic cell score is used as an indicator trait for CM as the genetic correlation is around 0.7 (Lund *et al.* 1999; Carlén *et al.* 2004; Heringstad *et al.* 2006), and SCS is easier to record. It is expected that many of the genes affecting SCS also affect CM; however, it is not known if QTL that have been identified for SCS also affect CM. Secondly, the QTL have only been detected by within-family linkage analysis (LA) studies. Such marker associations can only be used for selection within particular families, as the linkage phase with flanking markers can vary between families. It is difficult to implement MAS using markers that are only informative within families, and therefore MAS can be practised on a limited scale (Dekkers 2004). Using a combined linkage disequilibrium and linkage analysis (LDLA), QTL can potentially be mapped to a region less than 1 cM using closely linked markers (Meuwissen & Goddard 2000). This approach would identify haplotypes with predictive ability in the general population using markers within the linkage disequilibrium (LD) or LDLA confidence intervals.

Lund *et al.* (2007) detected a QTL for CM on bovine chromosome 9 (BTA9) using LA in a joint analysis of three Nordic cattle breeds, Swedish Red and White (SRB), Finnish Ayrshire (FA) and Danish Red (DR). However, the confidence interval for the QTL position was too large for potential use in selection. These three Nordic red breeds are distinct but with historic and recent genetic links. Together they form a powerful resource for fine-mapping QTL using the LDLA mapping approach. The same alleles are likely to be segregating in each breed, but the common founder of a given genetic variant is likely to be many generations from the current population. Using this approach, greater precision of the QTL positions should be obtained because of the large number of meioses separating the breeds.

When a QTL has a pleiotropic effect on two or more traits, a joint analysis involving all the traits may give a higher statistical power of detection, and a higher precision of the estimated map position compared with an analysis using the traits individually (Jiang & Zeng 1995; Knott & Haley 2000; Sørensen *et al.* 2003). This is especially true when a second correlated trait with higher heritability is used together with a trait with lower heritability (Sørensen *et al.* 2003). It was therefore expected that using SCS as a trait correlated with CM would increase the precision with which the QTL could be mapped. Furthermore, multi-trait QTL mapping allows decomposition of variances and covariances into polygenic and QTL components, which is also important for the application of the QTL in selection programmes.

QTL affecting milk, fat and protein yields have also been reported segregating on BTA9 (Georges *et al.* 1995; Vilkki *et al.* 1997; Zhang *et al.* 1998; Wiener *et al.* 2000). Vilkki *et al.* (1997) detected QTL affecting milk and protein yields on BTA9 in FA cattle. It was therefore interesting to test if the QTL affecting mastitis incidence segregating on BTA9 also affects yield traits. There are also well-established associations between mastitis and several udder type traits (Rupp & Boichard 1999, 2003). Analysing possible pleiotropic effects of mastitis, QTL on udder-type traits will help to understand if there is an underlying genetic relationship between them. Therefore, objectives of the QTL fine-mapping study reported here were (1) to fine-map the QTL on BTA9 affecting CM and/or SCS using combined LDLA within and across populations, (2) to identify the haplotypes associated with variations in mastitis resistance and (3) to study possible pleiotropic effects of mastitis QTL on yield and udder conformation traits.

## Materials and methods

### Animals

Animals in this study belonged to the three Nordic cattle breeds DR, FA and SRB. The largest families with progeny-tested sons were selected. A total of 34 families consisting of 9 grandsire families with 287 sons from DR, 11 grandsire families with 420 sons from FA and 14 grandsire families with 372 sons from SRB were studied using a granddaughter design (Weller *et al.* 1990). The families included between 16 and 74 progeny-tested sons. Only the paternal half-sib relationship was considered in the pedigree.

### Traits

The udder health traits analysed were CM and SCS. Three yield traits (milk, fat and protein yields) were considered along with eight udder conformation traits [dairy form, fore udder attachment, rear udder height, udder cleft, udder depth, teat placement (front), teat placement (back) and teat length]. Although the phenotypes for CM, SCS and milk yield traits were available for most of the genotyped individuals, the phenotypes for udder conformation traits were available for only some of the animals. The numbers of individuals available with records for udder conformation traits were 337 for FA, except udder form (107) and teat placement (105); 290 in DR, except udder height (99) and teat placement (99); and only 135 records for all udder conformation traits in SRB. The phenotypes for CM and SCS were derived from the models used to produce breeding values in the individual countries (details in Lund *et al.* 2007). The phenotypes were standardized to the same mean and variation before across-population QTL analyses were performed. Phenotypes for the milk yield and udder conformation traits were derived by a joint evaluation model used in Nordic countries (Mäntysaari *et al.* 2006).

### Linkage map construction

A total of 67 microsatellite and SNP markers covering all of chromosome 9 (BTA9) were genotyped on all animals. Out of these, 45 were microsatellite markers selected from the USDA-MARC map (http://www.marc.usda.gov/genome/genome.html), and 22 were new markers developed within the project (Hastings *et al.*, in preparation).

Microsatellite loci were amplified in 10-μl PCRs containing 15 ng genomic DNA extracted from frozen semen samples, 10 pmol of each forward and reverse primer (forward primers were labelled with fluorescent tags), 2 mm dNTPs, 1 mm MgCl_2_ and 0.5 U *Taq* polymerase (AmpliTaq Gold polymerase; Applied Biosystems). The PCR protocol was 40 cycles with touchdown consisting of 45 s at 94 °C, 45 s at 65 to 52 °C and 1 min at 72 °C. PCR products were analysed for fragment length using ABI3700 or MegaBace1000 sequencers and genescan and genetic profiler software respectively. SNP genotyping was performed by KBiosciences using a proprietary primer-extension method.

Marker order and map distances were estimated using crimap 2.4 software (Green *et al.* 1990). The linkage map covers 96.4 cM of BTA9 (the markers and the linkage map are given in Table S1 and Fig. S1 respectively). The distance between markers mapped at the same location was set to 0.05 cM for the QTL analysis.

### Linkage phase construction

The first step of the analysis was to infer linkage phases of the marker alleles of the genotyped animals to construct their paternally and maternally inherited marker haplotypes. The phase of the paternally and maternally inherited chromosomes was constructed using gdqtl software (B. Guldbrandtsen, personal communication), which is a likelihood-based method developed for granddaughter design QTL studies. The phases of the grandsires’ chromosomes were also inferred. After phasing, the marker data consisted of 2*s* sire chromosomes, *n* paternally inherited chromosomes of the sons and *n* maternally inherited chromosomes of the sons, where *s* and *n* correspond to the number of sire families and the number of sons in the design respectively.

### QTL analysis

The data analysis was carried out in three steps. First, single trait (ST) models for CM and SCS including LA (ST_LA_), LD (ST_LD_) and combined LDLA (ST_LDLA_) were used within each breed, as well as across combinations of breeds. Second, two-trait models (including CM and SCS) were used to quantify if a pleiotropic QTL model (MT_P_) or a two-linked QTL model (MT_L_) was the most probable. Third, two-trait models were analysed with a pleiotropic QTL affecting CM and one of the correlated traits, i.e. one of the milk production traits or one of the udder conformation traits. The third step was carried out to examine if the QTL for CM segregating on BTA9 (Lund *et al.* 2007) had an effect on these correlated traits.

The pleiotropic and linked QTL models can be written as:

(1)
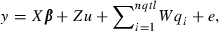


where ***y*** is an *n* × *t* vector of observations on *t* = {1, 2} traits, ***X*** is a design matrix, ***β*** is a vector of fixed effects, ***Z*** is a matrix relating records to individuals, ***u*** is a vector of additive polygenic effects, ***W*** is a matrix relating the record of the QTL effect to each individual, ***q***_*i*_ is a vector of additive QTL effects corresponding to the *i*th QTL and ***e*** is a vector of residuals. The number of QTL, *nqtl*, is assumed to be equal to one or two. The random variables *u*, *q* and *e* are assumed to be multivariate normally distributed and mutually uncorrelated. Specification of pleiotropic and linked QTL models can be seen in Lund *et al.* (2003).

### Estimation of parameters

The variance components were estimated using the average information restricted maximum-likelihood algorithm (Jensen *et al.* 1997), as implemented in the software package dmu (Madsen *et al.* 2006). The restricted likelihood was maximized with respect to the variance components associated with the random effects in the model. Maximizing a sequence of restricted likelihoods over a grid of specific positions yields a profile of the restricted likelihood for the QTL position. The parameters were estimated at the mid-point of each marker bracket along the chromosome. The fraction of the total additive genetic variance explained by the QTL was estimated as 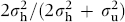, where 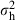 and 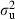 correspond respectively to the variance component associated with the haplotypes effect and the additive polygenic effect.

### Estimation of IBD probabilities

#### Linkage analysis

The identical-by-descent (IBD) probabilities between QTL alleles of any two founder haplotypes (Hs and Hm) were assumed to be zero, i.e. founder haplotypes were unrelated (Meuwissen *et al.* 2002). The probability of inheriting the paternal or maternal allele from the sire at the putative QTL and the IBD matrix was computed using a recursive algorithm (Wang *et al.* 1995). The IBD matrices were computed for every 2 cM interval along the chromosome and used in the subsequent variance component estimation procedure.

#### Combined linkage disequilibrium and linkage analysis

In the combined LDLA analysis, the IBD probabilities between QTL alleles of any two founder haplotypes were calculated using the method described by Meuwissen & Goddard (2001). The number of generations since the founder generation and the effective population size were both set to 100. Windows of 10 markers (five markers at the left and five markers at the right of the putative position) were considered in order to compute the IBD probabilities. Different size marker windows of e.g. six markers, four markers etc. were used to calculate IBD probabilities within the region of the LDLA peak to examine if fewer markers were sufficient to explain the QTL variance detected by the 10-marker haplotypes. Founder haplotypes were grouped into distinct clusters. We used (1 − IBD_ij_) as a measure of distance between the *i*th and *j*th haplotypes. A cluster was defined as a group of haplotypes that coalesce into a common node (IBD > 0.90). Haplotypes within a cluster were assumed to carry identical QTL alleles (IBD probability = 1.0) whereas haplotypes from different clusters were assumed to carry distinct QTL alleles and were therefore considered to be independent (IBD probability = 0). The IBD matrices were computed at the midpoints of each marker interval and used in the subsequent variance component estimation procedure.

#### Test statistics

Hypothesis tests for the presence of QTL were based on the asymptotic distribution of the likelihood ratio test (LRT) statistic, LRT = −2ln(*L*_reduced_ − *L*_full_), where *L*_reduced_ and *L*_full_ were the maximized likelihoods under the reduced and full models respectively. For the one-QTL model, the reduced model excluded the QTL effect being analysed. The two-linked QTL (each affecting one of the traits) hypothesis was compared with the no-QTL hypothesis or the one-QTL (affecting one of the two traits) hypothesis. Thresholds were calculated using the method presented by Piepho (2001). This is a quick method for computing approximate threshold levels that control the chromosome-wide and genome-wide type I error rates of tests for QTL. This method is a computationally inexpensive alternative to permutation procedures. It requires the values of the LRT from each of the putative QTL positions along the chromosome, the number of chromosomes, the degrees of freedom (df) for the LRT (df = number of parameters of *H*_full_– number of parameters of *H*_reduced_) and the type I error rate (*α*).

## Results

### Single-trait QTL analyses for CM and SCS

#### Danish Red

The ST_LDLA_ and ST_LD_ models identified a QTL at 74.1 cM (Tables 1 and 2). This QTL explained 44% of the genetic variance for CM in the ST_LDLA_ model. By default a 10-marker window was used to estimate the IBD probabilities. As the ST_LDLA_ and ST_LD_ QTL peaks for CM were in a narrow region, a four-marker window was used to estimate the IBD probabilities. The four-marker haplotype analysis had the highest QTL peak for CM between markers *INRA144* and *INRA084*. The SCS QTL identified by the ST_LDLA_ and ST_LD_ models was located at 39.8 cM (Tables 1 and 2). This QTL explained 31% of the total genetic variance in SCS in the ST_LDLA_ model.

**Table 1:** Summary of across-family linkage disequilibrium and linkage analysis (LDLA) using the variance component method.

Breed	Trait	Position (cM)	Peak LRT statistics	Marker interval
Danish Red (DR)	CM	74.1	8.5*	*BM2819–INRA144*
	SCS	39.8	16.9**	*DIK2741–TGLA261*
Finnish Ayrshire (FA)	CM	73.9	5.4	*BM4208–BMS2819*
	SCS	38.1	7.3*	*DIK2810–DIK5364*
Swedish Red and White (SRB)	CM	67.2	5.6	*BMS1724–DIK2145*
	SCS	74.1	5.7	*BMS2819–INRA144*
DR + FA	CM	74.1	9.9*	*BMS2819–INRA144*
	SCS	38.1	9.6*	*DIK2810–DIK5364*
FA + SRB	CM	77.2	13.4*	*INRA084–ESR1*
	SCS	95.1	10.4*	*BMS1943–BMS1967*
DR + FA + SRB	CM	73.9	14.9**	*BM4208–BMS2819*
	SCS	95.1	8.5*	*BMS1943–BMS1967*

* *P*<0.05.

** *P*<0.01.

**Table 2:** Summary of across-family linkage disequilibrium (LD) analysis using the variance component method.

Breed	Trait	Position (cM)	Peak LRT statistics	Marker interval
Danish Red (DR)	CM	74.1	13.6**	*BM2819–INRA144*
	SCS	39.8	13.0**	*DIK2741–TGLA261*
Finnish Ayrshire (FA)	CM	73.9	3.6	*BM4208–BMS2819*
	SCS	–	<1.0	*–*
Swedish Red and White (SRB)	CM	33.7	4.3	*DIK3002–DIK3003*
	SCS	46.4	2.2	*BM4204–DIK4926*
DR + FA	CM	74.1	5.5	*BMS2819–INRA144*
	SCS	39.8	2.0	*TGLA261–ILSTS013*
FA + SRB	CM	73.9	5.4	*BM4208–BMS2819*
	SCS	37.0	1.7	*BMS817–BMS555*
DR + FA + SRB	CM	73.9	8.9*	*BM4208–BMS2819*
	SCS	89.4	2.3	*PLG–IGF2R*

* *P*<0.05.

** *P*<0.01.

#### Finnish Ayrshire

A suggestive QTL for CM was located between 58 and 82 cM (one-LOD support interval; Lynch & Walsh 1998) in the ST_LA_ analysis (Table 3). The highest LRT for the CM QTL was at 73.9 cM using both the ST_LDLA_ and the ST_LD_ models but this QTL did not exceed the 5% significance threshold (Tables 1 and 2). For SCS the highest ST_LDLA_ LRT peak was at 38.1 cM (Table 1). The QTL explained 18% of the total genetic variance in SCS in the ST_LDLA_ model.

**Table 3:** Summary of across-family linkage analysis (LA) using the variance component method.

Breed	Trait	Position (cM)	Peak LRT statistics	Marker interval
Danish Red (DR)	CM	46.4	4.4	*BM4208–DIK4926*
	SCS	44.2	4.2	*DIK4720–BM4204*
Finnish Ayrshire (FA)	CM	68.2	4.2	*BM7209–SLU2*
	SCS	37.0	5.1	*BMS817–BMS555*
Swedish Red and White (SRB)	CM	67.4	9.9*	*DIK2145–BM7209*
	SCS	73.1	6.1	*BM7234–BM4208*
DR + FA	CM	68.2	3.2	*BM7209–SLU2*
	SCS	37.0	6.6*	*BMS817–BMS555*
FA + SRB	CM	68.2	15.3**	*BM7209–SLU2*
	SCS	95.1	9.9*	*BMS1943–BMS1967*
DR + FA + SRB	CM	68.2	13.5**	*BM7209–SLU2*
	SCS	95.1	8.5*	*BMS1943–BMS1967*

* *P*<0.05.

** *P*<0.01.

#### Swedish Red and White

The ST_LA_ model for CM was significant (*P*< 0.05) with the highest LRT at 67.4 cM (Table 3). The QTL interval was large, spanning 59–81 cM (one-LOD support interval). The QTL explained 23% of the genetic variance in CM. The highest ST_LA_ LRT for SCS QTL was at 73.1 cM but it did not reach the 5% significance threshold (Table 3). Although there was evidence for linkage, no significant LDLA or LD peaks were observed for either of these two traits in the single-trait analysis in SRB (Tables 1 and 2).

#### Across-breeds analysis

**Figure 1:**
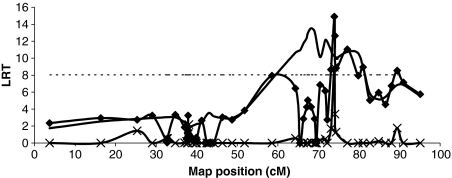
QTL profiles (— LA; ♦ LDLA; × LD; 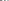
5% significant threshold) for the clinical mastitis showing an LDLA/LD peak between the markers *BM4208* and *INRA144* in combined analyses of Finnish Ayrshire, Danish Red and Swedish Red and White cattle.

All three models, ST_LA_, ST_LDLA_ and ST_LD_, detected QTL affecting CM segregating on BTA9 when the combined data from three breeds were analysed (Tables 1–3). These results were in agreement with the analyses within individual breeds, and gave a narrow LDLA/LD peak for CM at 73.9 cM (Tables 1 and 2; Fig. 1). The QTL interval using the ST_LA_ model spanned the region from 59 to 81 cM. In this interval, there was more than one peak when LDLA analysis was applied. However, fitting a two-QTL model with one QTL fixed at 73.9 cM and searching for the second QTL did not provide evidence of another linked QTL for CM segregating in this chromosomal region. The highest LRT for the SCS QTL was at 95.1 cM in both the ST_LA_ and ST_LDLA_ models (Tables 1 and3). The ST_LDLA_ model gave an additional peak for SCS at 74.1 cM between markers *BM2819* and *INRA144*, close to the CM QTL location. The QTL explained 18% and 17% of the total genetic variance for CM and SCS respectively.

**Figure 2:**
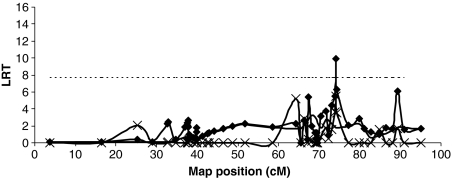
QTL profiles (— LA; ♦ LDLA; × LD; 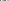
5% significant threshold) for the clinical mastitis showing an LDLA/LD peak between the markers *BMS2819* and *INRA144* in combined Finnish Ayrshire and Danish Red cattle.

**Figure 3:**
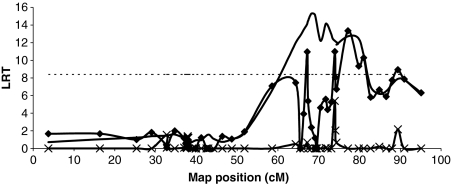
QTL profiles (— LA; ♦ LDLA; × LD; 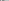 5% significant threshold) for the clinical mastitis showing an LDLA/LD peak in combined Finnish Ayrshire and Swedish Red and White cattle.

The ST_LDLA_ LRT peaks for CM QTL in DR and FA were only 0.2 cM apart in the within-breed analyses. Therefore, combined analyses of these two breeds were carried out (Fig. 2). The ST_LDLA_ analyses identified a QTL for CM at 74.1 cM (Table 1). When ST_LDLA_ analysis for CM with four-marker haplotypes was used to calculate the IBD probabilities, the highest LRT was at the same position as observed with the 10-marker haplotype analysis. The combined data from FA and SRB were also jointly analysed, because these two breeds are closely related (Holmberg & Andersson-Eklund 2004). In the ST_LA_ analysis, the QTL interval (one-LOD support) for CM was between 64 and 81 cM with the peak LRT at 68.2 cM (Table 3; Fig. 3). The ST_LDLA_ profile for CM had three peaks, the highest at 77.2 cM. However, evidence of LD was only around 73.9 cM. Single-trait analysis of SCS with ST_LA_ and ST_LDLA_ in combined FA and SRB had the highest LRT at 95.1 cM (Tables 1 and 3). Although the LRT for both the ST_LA_ and ST_LDLA_ models for SCS were significant, no LD was observed at this position.

### Clustering of the founder haplotypes

**Figure 4:**
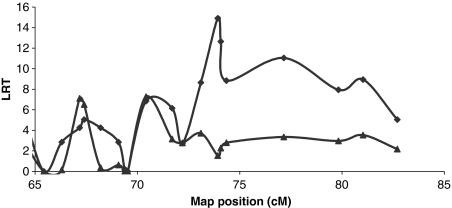
The QTL profiles with the four-marker haplotype (*BM4208–BMS2819–INRA144–INRA084*) as a fixed effect (▴) and the QTL profile without the haplotype in the model (♦).

The haplotypes with IBD probabilities greater than 90% were clustered together assuming that they contain the same allele at the putative QTL positions. The LDLA and LD analyses suggested that a QTL is segregating for CM around 74 cM within the marker haplotype *BM4208–BMS2819–INRA144–INRA084*. This four-marker haplotype, when included as a fixed effect in the model, explained the QTL variance for CM at this chromosomal location (Fig. 4). This adds further support that this haplotype contains a QTL for mastitis resistance. Therefore, the clustering of founder haplotypes and haplotype effects around the midpoint between the markers *BMS2819* and *INRA144* was studied further. This was done within breeds and also across the three red breeds (Table 4). In the across-breed analyses, haplotypes from FA and SRB clustered together more frequently than the DR haplotypes did with haplotypes from FA or SRB. The frequency of the large clusters ranged from 12% to 30% in different populations. Table 5 presents the allelic combinations and effects on CM for haplotypes with high frequencies in the combined three populations. Effects are expressed in standard deviation units of breeding value.

**Table 4:** Summary of clustering of haplotypes at 74.1 cM, the midpoint between *BMS2819* and *INRA144*, within breeds and across breeds.

	No. of founder haplotypes					
Breed	Dam origin	Grandsire origin	Total	No. of clusters	No. of clusters with frequency >5%	Frequency of the largest cluster
DR	287	18	305	54	5	0.12
FA	420	22	442	44	9	0.14
SRB	372	28	400	48	7	0.30
DR + FA	707	40	747	85	3	0.11
FA + SRB	792	48	840	76	7	0.21
DR + FA + SRB	1079	66	1145	92	6	0.18

**Table 5:** The allelic combination of the four-marker haplotype (*BM4208–BMS2819–INRA144–INRA084*) for the large clusters and their effects on mastitis resistance in combined data from three breeds.

	No. of haplotypes in the cluster					
Allelic combination	Dam origin	Grandsire origin	Total	Haplotype frequency	Grandsires haplotypes in the cluster	Haplotype effect ± SEP^*^*
171-129-199-104/106	38	3	41	0.04	2 FA + 1 SRB	+0.24 ± 0.10
173-146-191-108	59	2	61	0.05	2 SRB	+0.21 ± 0.10
167-119-199-102/106/108	46	2	48	0.04	2 FA	+0.15 ± 0.10
167-119-212-108	196	9	205	0.18	2 FA + 7 SRB	+0.03 ± 0.06
169-129-201-100/106/110	67	2	69	0.06	4 SRB + 1 DR	−0.003 ± 0.09
167-112-199-96/102/106	75	6	81	0.07	3 FA + 2 SRB + 1 DR	−0.04 ± 0.08
173-142-206-108	52	4	56	0.05	2 FA + 2 SRB	−0.08 ± 0.10
169-129-199-102/106	40	4	44	0.04	4 DR	−0.17 ± 0.10
163-123-185-100	52	6	58	0.05	2 FA + 4 SRB	−0.20 ± 0.09

* SEP,standard error of prediction;

‘+’ indicates positive effect on mastitis resistance and ‘−’ indicates negative effect on mastitis resistance. The effects of haplotypes are expressed in standard deviation units of breeding values standardized to (0,1).

### Multi-trait analysis

#### Clinical mastitis and SCS

**Figure 5:**
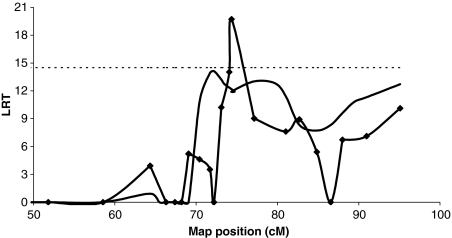
QTL profiles (— LA; ♦ LDLA; 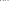 5% significant threshold) for the two-trait single QTL model affecting clinical mastitis and somatic cell score in combined breed data.

Multi-trait analysis of CM and SCS was carried out using both MT_P_ and MT_L_ models in the combined data from three breeds (Table 6; Fig. 5). The combined data with an MT_P_ model gave an LDLA peak with high LRT at 74.4 cM, between markers *INRA144* and *INRA084*. This QTL explained 42% and 14% of the genetic variation in CM and SCS respectively. The correlation between the QTL effects for these two traits was estimated to be 0.72. A LA with the MT_L_ model in the three breeds positioned the CM QTL at 79.7 cM and the SCS QTL at 95.1 cM. When LDLA analysis was carried out with a 2-trait model with QTL affecting CM and not SCS (MT_CM_), the highest LRT (12.0) was at 74.4 cM between *INRA144* and *INRA084*. In a similar 2-trait model, when the QTL was fitted to affect SCS only and not CM (MT_SCS_), the peak disappeared.

**Table 6:** Summary of the two-trait analyses with a pleiotropic QTL model affecting both CM and SCS vs. the two-linked QTL model, each affecting one trait.

	Pleiotropic QTL				Two-linked QTL				
	LA		LDLA		LA			LDLA	
Breed	QTL position (cM)	LRT	QTL position (cM)	LRT^1^^1^	CM QTL (cM)	SCS QTL (cM)	LRT^1^^1^	SCS QTL when CM QTL fixed at 74.1 cM	LRT^2^^2^
DR	3.7	8.1	69.1	10.6	95.1	3.7	7.2	Did not converge	–
FA	34.7	4.6	48.7	6.5	71.7	37.5	5.5	72.2	0.15
SRB	73.1	12.4	77.2	11.6	70.4	73.5	10.2	74.1	4.37
DR + FA + SRB	72.2	14.1	74.4	19.7**	79.7	95.1	14.1	74.1	5.1

LRT, likelihood ratio test.

1 Likelihoods were compared with a hypothesis of no QTL.

2 Likelihoods were compared with the clinical mastitis QTL fixed at 74.1 cM, and the search for SCS QTL was restricted to between 59 and 80 cM on BTA9.

** *P*<0.01.

The LDLA analysis with an MT_L_ model was carried out with the CM QTL fixed at 74.1 cM and the chromosomal region searched for SCS QTL. This search was restricted to between 59 and 80 cM of BTA9 to examine if an additional QTL affecting SCS linked to the CM QTL is segregating in this region of BTA9 (Table 6). The MT_L_ did not provide evidence for another closely linked QTL for SCS in the vicinity of the CM QTL.

#### Mastitis and yield traits

The single-trait analyses (milk, protein and fat yields) and two-trait analyses (one trait being CM and the other one being one of the yield traits) indicated that there was a QTL affecting yield traits segregating on BTA9 in the same chromosomal region as the CM QTL in DR (results not shown). However, there was not enough power in this study to distinguish if the QTL affecting CM had a pleiotropic effect on yield traits or if there were two linked QTL, one affecting CM and the other one affecting yield. None of the MT_P_ models with CM or any of the three yield traits showed any significant pleiotropic QTL on BTA9 in FA and SRB.

### Mastitis and udder type traits

Eight udder conformation traits were studied. Across-family single-trait analyses did not provide evidence of QTL affecting udder conformation traits on BTA9 in any of the breeds. The MT_P_ model with one udder conformation trait and CM did not give any evidence that the CM QTL segregating on BTA9 had a pleiotropic effect on any of the udder conformation traits considered in the study. Our search for the QTL affecting udder conformation traits was limited by the restricted availability of the phenotypic records.

## Discussion

In this paper, we describe the localization of a QTL affecting CM to an interval less than 1 cM on BTA9 between markers *BM4208* and *INRA084* using data from three Nordic red cattle breeds. This QTL was evident in both within- and across-breed analyses. The strongest evidence for LD was in DR and FA cattle, and it was also supported by across-breed analyses. A QTL affecting CM and SCS was initially found on BTA9 in the joint analysis of three Nordic breeds using LA methods (Lund *et al.* 2007). However, in that study the location of the QTL had a confidence interval (one-LOD support) spanning 21 cM. Because the location of the QTL differed between the three breeds, it was not clear whether or not the same QTL was segregating in the three populations, which have both historic and recent genetic links. In order to refine the QTL location, additional markers were genotyped, including newly generated markers. In total 67 markers were included in the linkage map, although several of these were very closely linked and mapped to the same location, as there was an insufficient number of recombinations between them to place them in a specific order. Overall 29 markers were mapped within a 20-cM region spanning the QTL one-LOD support confidence interval, giving an average marker density of 0.7 cM in this region. We used a combined LDLA approach that has proven to be powerful for fine-mapping QTL, taking advantage of the structure of the dairy cattle population (Grisart *et al.* 2002; Meuwissen *et al.* 2002). Within- and across-population QTL analyses using LDLA models narrowed down the QTL location to a four-marker haplotype. When the haplotype was considered as a fixed effect in the ST_LDLA_ model, the evidence for the QTL disappeared. This means that the QTL variance in CM was fully explained by this haplotype and confirms the location of the CM QTL within the four-marker haplotype or in very strong LD with it. The ability to narrow the QTL region and increase the statistical support for the QTL demonstrates the advantage of QTL mapping using more than one population that have close and/or historic genetic links. The evidence for QTL affecting CM was not strong when analysed within a breed and the QTL location spanned more than 20 cM. However, joining the data from three breeds, the QTL confidence interval decreased significantly, to less than 1 cM. In addition as the QTL was detected in more than one breed, there was increased confidence in its existence and position.

The SRB breed is closely related with FA cattle (Holmberg & Andersson-Eklund 2004). The clustering of haplotypes in our data supported the known breed history. The FA and SRB haplotypes clustered together more often than haplotypes of either of these breeds did with DR haplotypes. There was evidence of LD at the QTL position in the combined-breed analysis. These observations suggest that the QTL on BTA9 detected in the three breeds affecting mastitis most likely has a common origin. A large number of haplotypes were observed because of the use of variable microsatellites and the supposedly long time since the common origin. Different haplotypes showing identical effects might carry the same QTL allele. Using a dense marker map within the region may help to reduce the number of associated haplotypes in these populations, i.e. to define the actual IBD segments associated with specific QTL alleles.

Boichard *et al.* (2003) reported a QTL affecting SCS in French dairy cattle, segregating on BTA9 close to the marker *BMS1967* at a position equivalent to 96.4 cM in our linkage map. This suggests that the QTL in French cattle is probably not the same as the one identified for CM in this study. However, there is evidence for another QTL for SCS at the distal end of BTA9, as reported in French cattle. This SCS QTL did not show any (pleiotropic) effect on CM in our study.

The QTL affecting yield traits were observed segregating in the CM QTL region in DR, but not in FA and SRB. It was not possible to distinguish whether there are two linked QTL, one affecting CM and the other affecting yield, or one pleiotropic QTL affecting both CM and yield traits in DR. Vilkki *et al.* (1997) identified a QTL on BTA9 affecting milk and protein yield in FA cattle, with the most probable location near the marker *UWCA9* (equivalent to 40.4 cM of our map). This QTL region also does not overlap with the mastitis QTL identified here. There were only three families in common between the Vilkki *et al.* (1997) study and the one reported here, which is the most likely reason that this milk yield QTL was not identified in the FA. Wiener *et al.* (2000) reported a QTL affecting milk and protein yield close to marker *BM4208*. This marker is within the four-marker haplotype affecting CM found in the present study. Georges *et al.* (1995) and Zhang *et al.* (1998) both reported QTL affecting milk and protein yields close to the marker *TGLA73* (63.4 cM in our map), again close to the QTL position identified in this study.

Favourable associations have been reported between mastitis resistance and several udder type traits e.g., higher and more tightly attached udders are associated with lower SCS and lower CM (Rupp & Boichard 1999, 2003). Therefore, genes affecting udder conformation may also affect mastitis incidence. However, no QTL were found on BTA9 for any of the eight udder conformation traits using either a single-trait analysis or the multi-trait analysis with CM. One reason for this result may be the incomplete availability of the udder-type phenotypic records for the individuals used in this study. On the other hand, the result may reflect different risk factors for mastitis: udder conformation may be associated with the physical contamination by the causative bacteria, while the QTL study may localize the genes involved in the immunological control of infection.

In conclusion, a QTL affecting CM was fine-mapped on BTA9 in three Nordic red cattle breeds. A four-marker haplotype containing the QTL was identified at 74.1 cM. The haplotypes associated with variations in mastitis resistance were identified and could be used in MAS programmes. This QTL also appears to be associated with milk and protein yield in DR, but did not show any association with yield traits in FA and SRB.

## Supplementary material

The following supplementary material is available for this article onlinefrom http://www.blackwell-synergy.com/doi/full/10.1111/j.1365-2052.2008.01729.x

**Figure S1** Linkage map of BTA9.

**Table S1** Markers included in the linkage map.
