# Supplementary figures and images for: Fine-mapping QTL for mastitis resistance on BTA9 in three Nordic red cattle breeds
Source: Anim Genet. 2008 Aug;39(4):354–62. doi: 10.1111/j.1365-2052.2008.01729.x (PMC2655356; doi:10.1111/j.1365-2052.2008.01729.x)

**Figure S1** Linkage map of BTA9.

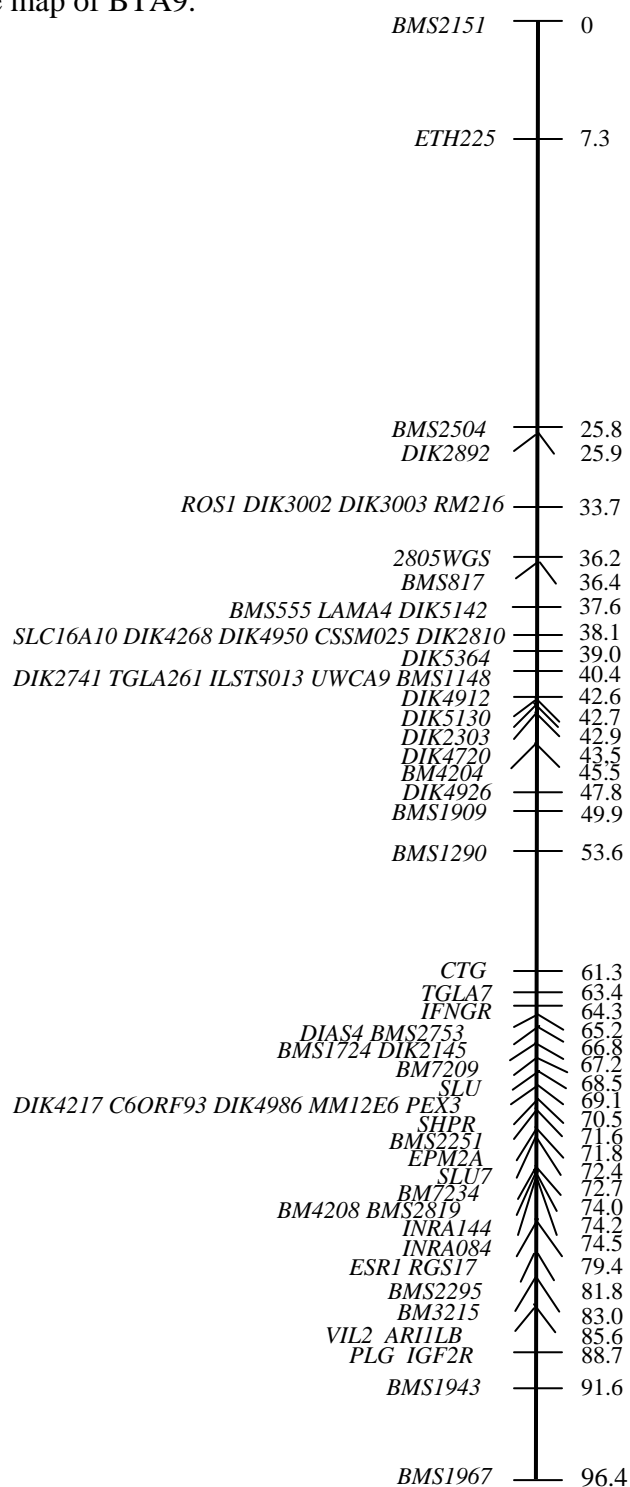

Supplement: Supplementary file 1 [file age0039-0354-SD1.pdf]
