# Supplementary material for: Fine-mapping QTL for mastitis resistance on BTA9 in three Nordic red cattle breeds
Source: Anim Genet. 2008 Aug;39(4):354–62. doi: 10.1111/j.1365-2052.2008.01729.x (PMC2655356; doi:10.1111/j.1365-2052.2008.01729.x)
Supplement: Supplementary file 2 [file age0039-0354-SD2.pdf]

**Table S1** Markers included in the linkage map.

| Marker | Marker type | Sequence accession no. |
|---|---|---|
| *BMS2151* | microsatellite | USDA-MARC* |
| *ETH225* | microsatellite | USDA-MARC |
| *BMS2504* | microsatellite | USDA-MARC |
| *DIK2892* | microsatellite | USDA-MARC |
| *ROS1* | SNP | BZ928122 |
| *DIK3002* | microsatellite | USDA-MARC |
| *DIK3003* | microsatellite | USDA-MARC |
| *rm216* | microsatellite | USDA-MARC |
| *2805WGS131* | SNP | GI53654756 |
| *BMS817* | microsatellite | USDA-MARC |
| *BMS555* | microsatellite | USDA-MARC |
| *LAMA4* | SNP | BZ909732 |
| *DIK5142* | microsatellite | USDA-MARC |
| *SLC16A10* | SNP | BZ900643 |
| *DIK4268* | microsatellite | USDA-MARC |
| *DIK4950* | microsatellite | USDA-MARC |
| *CSSM025* | microsatellite | USDA-MARC |
| *DIK2810* | microsatellite | USDA-MARC |
| *DIK5364* | microsatellite | USDA-MARC |
| *DIK2741* | microsatellite | USDA-MARC |
| *TGLA261* | microsatellite | USDA-MARC |
| *ILSTS013* | microsatellite | L23487 |
| *UWCA9* | microsatellite | L15338 |
| *BMS1148* | microsatellite | USDA-MARC |
| *DIK4912* | microsatellite | USDA-MARC |
| *DIK5130* | microsatellite | USDA-MARC |
| *DIK2303* | microsatellite | USDA-MARC |
| *DIK4720* | microsatellite | USDA-MARC |
| *BM4204* | microsatellite | USDA-MARC |
| *DIK4926* | microsatellite | USDA-MARC |
| *BMS1909* | microsatellite | USDA-MARC |
| *BMS1290* | microsatellite | USDA-MARC |
| *CTGF* | SNP | NW_001495588.1 |
| *TGLA73* | microsatellite | USDA-MARC |
| *IFNGR1* | SNP | AAFC01159975 |
| *DIAS4* | microsatellite | AAFC1331944 |
| *BMS2753* | microsatellite | USDA-MARC |
| *TNF* | SNP | GI53462007 |
| *BMS1724* | microsatellite | USDA-MARC |
| *DIK2145* | microsatellite | USDA-MARC |
| *BM7209* | microsatellite | USDA-MARC |
| *SLU2* | microsatellite | AAFC01124035 |
| *DIK4217* | microsatellite | USDA-MARC |
| *C6ORF93* | SNP | NC_007307 |
| *DIK4986* | microsatellite | USDA-MARC |
| *MM12e6* | microsatellite | USDA-MARC |

| | | |
|---|---|---|
| *PEX3* | SNP | AAFC01018446 |
| *DEAD2* | SNP | GI53648076 |
| *SHPRH* | SNP | AAFC01645170 |
| *BMS2251* | microsatellite | USDA-MARC |
| *EPM2A* | SNP | AAFC01157463 |
| *SLU7* | microsatellite | AAFC01219686 |
| *BM7234* | microsatellite | USDA-MARC |
| *BM4208* | microsatellite | USDA-MARC |
| *BMS2819* | microsatellite | USDA-MARC |
| *INRA144* | microsatellite | USDA-MARC |
| *INRA084* | microsatellite | USDA-MARC |
| *ESR1* | SNP | AY538775.1 |
| *RGS17* | SNP | BZ921804 |
| *BMS2295* | microsatellite | USDA-MARC |
| *BM3215* | microsatellite | USDA-MARC |
| *VIL2* | SNP | gi27806350 |
| *ARID1B* | SNP | BZ853825 |
| *PLG* | SNP | NM173951 |
| *IGF2R* | SNP | NM174352 |
| *BMS1943* | microsatellite | USDA-MARC |
| *BMS1967* | microsatellite | USDA-MARC |

* http://www.marc.usda.gov/genome/genome.html
